# Supplementary material for: An open-source framework for large-scale, flexible evaluation of biomedical text mining systems
Source: J Biomed Discov Collab. 2008 Jan 29;3:1. doi: 10.1186/1747-5333-3-1 (PMC2276192; doi:10.1186/1747-5333-3-1)
Supplement: Additional file 1 — Evaluation platform source code. The additional file contains the software evaluation framework discussed in this paper. The most current release can be downloaded from SourceForge.net [1]. See the accompanying README file for instructions on installation and use. Also included in the distribution is a collection of UIMA wrappers for some commonly used BioNLP tools and annotated corpora. [file 1747-5333-3-1-S1.zip › bionlp_uima_repository-1.0.1/docs/api/src-html/edu/uchsc/ccp/uima/util/UIMA_Annotation_Util.html]

```
001    package edu.uchsc.ccp.uima.util;
002    
003    import java.util.regex.Matcher;
004    import java.util.regex.Pattern;
005    
006    import org.apache.uima.cas.CASException;
007    import org.apache.uima.jcas.tcas.Annotation;
008    
009    import edu.uchsc.ccp.uima.annotation.CCPTextAnnotation;
010    
011    public class UIMA_Annotation_Util {
012    
013        public static final boolean DEBUG = false;
014        
015       
016        
017        
018        /*
019         * This method removes blank lines from the beginning and end of the input paragraph
020         */
021        public static void removeLeadingAndTrailingBlankLines(Annotation annotation) {
022            Matcher matcher;
023            Pattern blankLineAtStartPattern = Pattern.compile("^(\\n+)");
024            
025            matcher = blankLineAtStartPattern.matcher(annotation.getCoveredText());
026            if (matcher.find()) {
027                if (DEBUG) {
028                    System.out.print("DEBUG -- UIMA_Annotation_Util.removeLeadingAndTrailingBlankLines: Removing blank line at start of annotation. Start index before removal: " + annotation.getBegin() + "  Start index after removal: ");
029                }
030                annotation.setBegin(matcher.end(1));
031                if (DEBUG) {
032                    System.out.println(annotation.getBegin());
033                }
034            }
035                    
036            Pattern blankLineAtEndPattern = Pattern.compile("(\\n+)$");
037            matcher = blankLineAtEndPattern.matcher(annotation.getCoveredText());
038            if (matcher.find()) {
039                if (DEBUG) {
040                    System.out.print("DEBUG -- UIMA_Annotation_Util.removeLeadingAndTrailingBlankLines: Removing blank line at end of annotation. End index before removal: " + annotation.getEnd() + "  End index after removal: ");
041                }
042                annotation.setEnd(matcher.start(1));
043                if (DEBUG) {
044                    System.out.println(annotation.getEnd());
045                }
046            }
047        }
048    
049        
050        /**
051         * trims spaces and &nbsp; from ends of a CCPTextAnnotation
052         * @param ccpTA
053         * @throws CASException
054         */
055       public static void trimCCPTextAnnotation(CCPTextAnnotation ccpTA) throws CASException {
056            /* trim beginning  */
057           int startIndex = 0;
058           String coveredText = ccpTA.getCoveredText();
059           String tempText = coveredText;
060           int endIndex = coveredText.length();
061           int count = 0;
062            while ((tempText.startsWith(" ") || tempText.startsWith("&nbsp;")) & count < 10) {
063                if (tempText.startsWith(" ")) {
064                    if (DEBUG) {
065                        System.err.println("Trimming space from beginning...");
066                    }
067                    startIndex++;
068                } else if (tempText.startsWith("&nbsp;")) {
069                    if (DEBUG) {
070                        System.err.println("Trimming &nbsp; from beginning...");
071                    }
072                    startIndex+=6;
073                } else {
074                    if (DEBUG) {
075                    System.err.println("neither space nor &nbsp; so the loop should break...");
076                    }
077                }
078                tempText = coveredText.substring(startIndex, endIndex);
079                if (DEBUG) {
080                    System.err.println("NEW TEMP TEXT =" + tempText + ";;");
081                }
082                count++;
083            }
084            
085            count = 0;
086            /* trim end */
087            while ((tempText.endsWith(" ") || tempText.endsWith("&nbsp;")) & count < 10) {
088                if (tempText.endsWith(" ")) {
089                    if (DEBUG) {
090                        System.err.println("Trimming space from end...");
091                    }
092                    endIndex--;
093                } else if (tempText.endsWith("&nbsp;")) {
094                    if (DEBUG) {
095                        System.err.println("Trimming space from end...");
096                    }
097                    endIndex-=6;
098                } else {
099                    if (DEBUG) {
100                        System.err.println("neither space nor &nbsp; so the loop should break...");
101                        }
102                }
103                tempText = coveredText.substring(startIndex, endIndex);
104                if (DEBUG) {
105                    System.err.println("NEW TEMP TEXT =" + tempText + ";;");
106                }
107                count++;
108            }
109            
110            int offset = ccpTA.getBegin();
111            UIMA_Util.setCCPTextAnnotationSpan(ccpTA, startIndex + offset, endIndex + offset);
112        }
113       
114    
115       public static boolean overlaps (CCPTextAnnotation ccpTA1, CCPTextAnnotation ccpTA2) {
116           
117           int s0start = ccpTA1.getBegin();
118           int s0end = ccpTA1.getEnd();
119           int s1start = ccpTA2.getBegin();
120           int s1end = ccpTA2.getEnd();
121           if ((s0start >= s1start & s0start < s1end) | (s0end > s1start & s0end <= s1end) | (s0start <= s1start & s0end > s1start) | (s0end >= s1end & s0start < s1end)) {
122               return true;
123           } else {
124               return false;
125           }
126           
127       }
128       
129    }
```
